# Lifetime risk of autosomal recessive neurodegeneration with brain iron accumulation (NBIA) disorders calculated from genetic databases

**DOI:** 10.1016/j.ebiom.2022.103869

**Published:** 2022-02-15

**Authors:** Hana Kolarova, Jing Tan, Tim M. Strom, Thomas Meitinger, Matias Wagner, Thomas Klopstock

**Affiliations:** a Department of Neurology, Friedrich-Baur-Institute, University Hospital, Ludwig Maximilian University of Munich, Ziemssenstraße 1a, Munich 80336, Germany; b Institute of Human Genetics, Technical University of Munich, Trogerstraße 32, Munich 81675, Germany; c Department of Pediatrics and Inherited Metabolic Disorders, First Faculty of Medicine, Charles University and General University Hospital, Ke Karlovu 2, Prague 12000, Czech Republic; d Department of Neurology, The Second Affiliated Hospital of Dalian Medical University, Dalian, China; e Institute of Neurogenomics, Helmholtz Zentrum Munich, Ingolstädter Landstraße 1, Neuherberg 85764, Germany; f LMU University Hospital, Department of Pediatrics, Dr. von Hauner Children's Hospital, Division of Pediatric Neurology, LMU Center for Development and Children with Medical Complexity, Ludwig-Maximilians-University, Munich, Germany; g German Center for Neurodegenerative Diseases (DZNE), Munich, Germany; h Munich Cluster for Systems Neurology (SyNergy), Munich, Germany

**Keywords:** Autosomal recessive NBIA disorders, Neurodegeneration, Lifetime risk, PKAN, PLAN, CoPAN

## Abstract

**Background:**

Neurodegeneration with brain iron accumulation (NBIA) are a group of clinically and genetically heterogeneous diseases characterized by iron overload in basal ganglia and progressive neurodegeneration. Little is known about the epidemiology of NBIA disorders. In the absence of large-scale population-based studies, obtaining reliable epidemiological data requires innovative approaches.

**Methods:**

All pathogenic variants were collected from the 13 genes associated with autosomal recessive NBIA (*PLA2G6, PANK2, COASY, ATP13A2, CP, AP4M1, FA2H, CRAT, SCP2, C19orf12, DCAF17, GTPBP2, REPS1)*. The allele frequencies of these disease-causing variants were assessed in exome/genome collections: the Genome Aggregation Database (gnomAD) and our in-house database. Lifetime risks were calculated from the sum of allele frequencies in the respective genes under assumption of Hardy-Weinberg equilibrium.

**Findings:**

The combined estimated lifetime risk of all 13 investigated NBIA disorders is 0.88 (95% confidence interval 0.70–1.10) per 100,000 based on the global gnomAD dataset (*n* = 282,912 alleles), 0.92 (0.65–1.29) per 100,000 in the European gnomAD dataset (*n* = 129,206), and 0.90 (0.48–1.62) per 100,000 in our in-house database (*n* = 44,324). Individually, the highest lifetime risks (>0.15 per 100,000) are found for disorders caused by variants in *PLA2G6, PANK2* and *COASY*.

**Interpretation:**

This population-genetic estimation on lifetime risks of recessive NBIA disorders reveals frequencies far exceeding previous population-based numbers. Importantly, our approach represents lifetime risks from conception, thus including prenatal deaths. Understanding the true lifetime risk of NBIA disorders is important in estimating disease burden, allocating resources and targeting specific interventions.

**Research in context**

**Evidence before this study**

Neurodegeneration with brain iron accumulation (NBIA) are a clinically and genetically heterogeneous group of inherited neurological disorders characterized by progressive extrapyramidal dysfunction and iron overload in specific brain areas. To date, epidemiological data are scarce, but all forms of NBIA are considered to be ultra-rare with a combined prevalence of 0.1–0.3 per 100,000, estimated based on reported cases. A population-genetic approach, using the allele frequencies of pathogenic *PANK2* variants, showed an estimated lifetime risk of panthothenate kinase-associated neurodegeneration of approx. 2 per 1,000,000.

**Added value of this study**

We utilized the publicly available Genome Aggregation Database comprising 125,748 exomes and 15,708 genomes, as well as our in-house database of 22,162 exomes in order to calculate the lifetime risk for all 13 autosomal recessive NBIA disorders. The combined lifetime risk estimate (due to the method as from conception) is up to 0.92 per 100,000. Moreover, we could rank all 13 disorders and define the disorders with the highest lifetime risk as being caused by mutations in *PLA2G6, PANK2*, and *COASY*.

**Implications of all the available evidence**

This study provides lifetime risk data for the group of autosomal recessive NBIA disorders using a population-based genotype approach. Our estimate of combined NBIA lifetime risk exceeds previous population-based epidemiological investigations by almost a magnitude. Understanding the actual lifetime risk of NBIA disorders is important for estimating disease burden, allocating resources and targeting specific interventions.

Alt-text: Unlabelled box

## Introduction

Neurodegeneration with brain iron accumulation (NBIA) comprises a group of rare inherited neurodegenerative diseases characterized by the eponymous abnormal iron accumulation in the brain, most pronounced in the basal ganglia. The hallmark clinical manifestations of NBIA disorders are extrapyramidal movement disorder, spasticity, developmental delay, dysarthria, ocular involvement and psychiatric symptoms.^1^ The disease course has a variable rate of progression, but in most cases leads to severe disability and premature death. As of today, treatment remains largely symptomatic, but a growing number of drugs are being tested in clinical trials.^2^

To date, 15 disorders have been identified as subtypes of NBIA.^3^ Their current classification links specific disease-causing genes to distinct cellular pathways: (i) Coenzyme A (CoA) biosynthesis (*PANK2, COASY*); (ii) lipid metabolism (*PLA2G6, c19orf12, FA2H, SCP2, CRAT*); (iii) autophagy (*WDR45, ATP13A2, AP4M1, REPS1*); (iv) iron homeostasis (*FTL, CP*) and (v) other, yet unknown mechanism (*GTPBP2, DCAF17*).^3^ All but two disorders ‒ X-linked ß-propeller-associated neurodegeneration (BPAN) caused by variants in *WDR45,* and autosomal dominant neuroferritinopathy caused by mutations in ferritin light chain (*FTL*) gene ‒ are inherited in an autosomal recessive manner.

Currently, only limited data exist on the epidemiology of NBIA disorders. For all NBIA forms, a combined prevalence of 0.1–0.3 per 100,000 has been estimated based on reported cases.^4^ Panthothenate kinase-associated neurodegeneration (PKAN) due to mutations in *PANK2* has generally been believed to be the most common form, estimated to account for 50–70% of all NBIA cases.^5^ However, the distribution of NBIA disorders may shift over time as patients with mutations in more recently recognized genes are increasingly reported.^6^ A study based on three different population-genetic models using the allele frequencies of pathogenic *PANK2* variants showed PKAN lifetime risk ranging from 1:396,006 in European, 1:480,826 in Latino, 1:523,551 in East Asian, 1:531,118 in South Asian and 1:1,526,982 in African populations.^7^ Estimated lifetime risk of mitochondrial membrane-associated neurodegeneration (MPAN) in Russia based on normalized allele frequency of the most common NM_001031726.3: c.204_214del; p.(Gly69Argfs*10) was 1:619,150.^8^ However, data on lifetime risks of other NBIA types are either limited to isolated populations^9^, ^10^, ^11^ or completely unknown.

By determining the population frequency of disease-causing alleles in our in-house database and the Genome Aggregation Database (gnomAD), we have previously assessed the lifetime risk of 249 autosomal recessive mitochondrial diseases and used data from newborn screening reports for three of these disorders and phenylketonuria as a proof of concept.^12^ Similarly, we now calculated the lifetime risk for all 13 autosomal recessive NBIA disorders known to date.

## Methods

The methodology was previously described in detail in.^12^ All raw data and variables that were used in any analyses have been uploaded to figshare (https://doi.org/10.6084/m9.figshare.16640281.v2).

### Ethics

The study was conducted within a research project approved by the local ethics committee of the Technical University of Munich (#5360/12S).

### In-house exome sequencing database

The in-house database of the Institute of Human Genetics (Munich, Germany) contains exome sequencing data of healthy unrelated individuals as well as patients with various genetic disorders. Exomes were sequenced as previously described using Agilent in solution exome enrichment kits, in >90% of samples Agilent Sure Select 50Mb v5 or Agilent Sure Select 60Mb v6 kits.^13^ In >98% of these samples, >98% of all coding regions of 13 NBIA genes were covered >20x, allowing sensitive variant detection.

### Defining the gene list

A set of 13 genes for autosomal recessive NBIA disorders was defined based on a literature review. The list of genes, their expected function and the associated monogenic disorders are shown in Table 1. Due to the different mode of inheritance, the lifetime risk of BPAN and neuroferritinopathy could not be assessed by our method.

**Table 1:** List of NBIA genes, their expected function and corresponding disorders.

Gene list	Function	Corresponding disorder	Acronym	MIM-ID
*PLA2G6*	lipid metabolism	Phospholipase A2-associated neurodegeneration	PLAN	# 610217
*PANK2*	coenzyme A biosynthesis	Panthothenate kinase-associated neurodegeneration	PKAN	# 234200
*COASY*	coenzyme A biosynthesis	COASY protein-associated neurodegeneration	CoPAN	# 615643
*ATP13A2*	autophagy	Kufor-Rakeb syndrome	KRS	# 606693
*CP*	iron homeostasis	Aceruloplasminemia	ACP	# 604290
*AP4M1*	autophagy	Spastic paraplegia 50, autosomal recessive	SPG 50	# 612936
*FA2H*	lipid metabolism	Fatty acid hydroxylase-associated neurodegeneration	FAHN	# 612319
*CRAT*	lipid metabolism	Neurodegeneration with brain iron accumulation 8	NBIA 8	# 617917
*SCP2*	lipid metabolism	Leukoencephalopathy with dystonia and motor neuropathy	LKDMN	# 613724
*C19orf12*	lipid metabolism	Mitochondrial membrane protein-associated neurodegeneration	MPAN	# 614298
*DCAF17*	unknown	Woodhouse-Sakati syndrome	WSS	# 241080
*GTPBP2*	unknown	Jaberi-Elahi syndrome	JES	# 617988
*REPS1*	autophagy	Neurodegeneration with brain iron accumulation 7	NBIA 7	# 617916
WDR45*	autophagy	ß-propeller-associated neurodegeneration	BPAN	# 300894
FTL**	iron homeostasis	Neuroferritinopathy	n/a	# 606159

All disorders are autosomal recessive except *X-linked and **autosomal dominant.

### Defining the set of pathogenic variants

First, we accessed the publicly available databases ClinVar (https://www.ncbi.nlm.nih.gov/clinvar/) and Human Gene Mutation Database (HGMD, http://www.hgmd.cf.ac.uk/ac/index.php), and collected all variants listed as “pathogenic” or “likely pathogenic” in at least one of these databases until January 2021. Additionally, our in-house exome database and the gnomAD (http://gnomad.broadinstitute.org/)^14^ were queried for loss of function (LoF) variants, including structural variants, not listed in any of the above databases and considered as being “pathogenic”. Variants in gnomAD whose LoF effect was questionable (e.g., presence only in a minor transcript, location in the last exon) were excluded. For variants with conflicting interpretation of pathogenicity in ClinVar, we reviewed the literature and evaluated the pathogenicity according to the criteria suggested by the American College of Medical Genetics and Genomics.^15^ Although these were not represented in gnomAD, a total of 13 *C19orf12* pathogenic variants that were reported in association with an autosomal dominant form of MPAN were removed from our analyses. For the 13 NBIA genes analysed here, we collected in total 1135 variants and later excluded 76 (7%) of them (as listed in Suppl. Table 3). Of note, some variants were described causing phenotypes different from the typical NBIA phenotype considered here, e.g. *ATP13A2* mutations associated with neuronal ceroid lipofuscinosis^16^ or *PANK2* mutations associated with hypobetalipoproteinemia, acanthocytosis and retinitis pigmentosa (HARP) syndrome, a condition allelic to PKAN.^17^ Since the phenotypes of these disorders largely overlap with the respective NBIA forms, we did not exclude these variants for our analyses.

### Assessment of allele frequencies

Two databases were utilized to assess the allele frequencies of disease-causing variants in the general population: (i) the v 2.1.1 variant data set of the gnomAD comprising 125,748 exomes and 15,708 genomes from unrelated individuals from different ethnic backgrounds; and ii) our in-house database. To prevent a selection bias in the analysis of our in-house database, we excluded NBIA patients (i.e., individuals with homozygous or compound heterozygous variants in NBIA genes) as well as their parents, leaving 22,162 exome datasets.

### Estimation of the lifetime risks of diseases

The lifetime risk, i.e. the cumulative lifetime incidence, is defined as the proportion of a population that will develop a disease at some point in life. The expected lifetime risk *R_i_* for an autosomal recessive NBIA disorder caused by mutations in gene *i* was calculated from the sum of the allele frequencies *q_ij_* (determined as described in 2.5) of the *n_j_* disease-causing variants (determined as described in 2.4) in the respective gene under the assumption of Hardy-Weinberg equilibrium and mutual independence of these rare variants. Biallelic combinations of variants were considered to be fully penetrant. Accordingly:

$$R_{i}={\left(q_{i1}+q_{i2}+\ldots +q_{in_{i}}\right)}^{2}={\left(\sum_{j=1}^{n_{i}}q_{ij}\right)}^{2}$$

The combined lifetime risk *R_total_* for developing one of the assessed NBIA disorders was calculated by summation of the lifetime risks of each of the diseases, assuming that these rare disorders are independent of each other and do not occur together.

$$R_{total}=\sum_{i}R_{i}=\sum_{i}{\left(\sum_{j=1}^{n_{i}}q_{ij}\right)}^{2}$$

Due to low numbers, 95% confidence intervals (95% CI) were calculated using the Clopper-Pearson Exact method.^18^

### Statistical analysis

Preplanned statistical analyses were performed using R 3.6.2. For correlations between numbers of disease-causing variants and the time period since the identification and association with an NBIA disorder (i.e. 2021 - the year of publication), the Spearman correlation coefficient was utilized. For the latter analysis, *p*-value of less than 0.01 was considered statistically significant due to the multivariate testing (Bonferroni correction for 5 hypotheses). The number of variants is listed as both non-adjusted and adjusted by a factor of gene size (number of amino acids for gene x/average number of amino acids of 13 NBIA genes).

### Role of funding sources

Funders (cf. Acknowledgments) had no role at all in study design, data collection, data analyses, interpretation, or writing of the report.

## Results

### Estimation of the lifetime risk of autosomal recessive NBIA disorders

The overall numbers of (likely) disease-causing variants and disease-causing alleles in 13 recessive NBIA genes, and associated lifetime risks of corresponding disorders according to both worldwide and European (non-Finnish) gnomAD population, and the in-house database are shown in Table 2 (including 95% confidence intervals). A more granular analysis of all eight subpopulations from the gnomAD dataset and the in-house database (including 95% confidence intervals) can be found in Supplementary Table 1. A detailed list of all variants and their associated allele frequencies in all datasets is available in the online material on figshare (https://doi.org/10.6084/m9.figshare.16640281.v2).

**Table 2:** Lifetime risk of 13 autosomal recessive NBIA disorders (including 95% confidence intervals).

Disease name	Gene	Number of disease-causing variants in gnomAD dataset	Number of disease-causing alleles in gnomAD dataset (worldwide)*	Number of disease-causing alleles in gnomAD dataset (European, Non-Finnish population)⁎⁎	Number of disease-causing variants In-house database	Number of disease-causing alleles In-house database⁎⁎⁎	Lifetime risk in worldwide population per 100,000 (gnomAD dataset; 95% CI)	Lifetime risk in European, non-Finnish population per 100,000 (gnomAD dataset; 95% CI)	Lifetime risk per 100,000 (in-house database)
**PLAN**	***PLA2G6***	108	288	124	34	75	0.26 (0.21–0.31)	0.30 (0.23–0.39)	0.29 (0.18–0.45)
**PKAN**	***PANK2***	79	343	121	25	54	0.24 (0.20–0.29)	0.15 (0.10–0.20)	0.15 (0.08–0.25)
**CoPAN**	***COASY***	44	306	152	12	41	0.15 (0.12–0.19)	0.19 (0.14–0.26)	0.09 (0.04–0.16)
**KRS**	***ATP13A2***	63	171	82	18	27	0.07 (0.06–0.09)	0.07 (0.05–0.10)	0.04 (0.02–0.08)
**ACP**	***CP***	57	110	53	19	34	0.04 (0.03–0.05)	0.06 (0.04–0.08)	0.06 (0.03–0.11)
**SPG 50**	***AP4M1***	40	101	61	14	23	0.02 (0.016–0.031)	0.05 (0.03–0.07)	0.03 (0.01–0.06)
**FAHN**	***FA2H***	27	69	43	11	11	0.02 (0.015–0.031)	0.03 (0.02–0.04)	0.006 (0.002–0.020)
**NBIA 8**	***CRAT***	42	90	33	14	28	0.02 (0.01–0.03)	0.01 (0.008–0.03)	0.04 (0.02–0.08)
**LKDMN**	***SCP2***	33	80	46	10	25	0.02 (0.012–0.025)	0.02 (0.01–0.04)	0.03 (0.01–0.07)
**MPAN**	***C19orf12***	21	84	55	10	33	0.01 (0.008–0.018)	0.03 (0.02–0.04)	0.06 (0.03–0.11)
**WSS**	***DCAF17***	29	49	33	9	47	0.01 (0.006–0.015)	0.02 (0.01–0.03)	0.11 (0.06–0.20)
**JES**	***GTPBP2***	14	31	13	2	10	0.003 (0.002–0.006)	0.003 (0.001–0.007)	0.005 (0.001–0.017)
**NBIA 7**	***REPS1***	14	14	5	1	5	0.0003 (0.0001–0.0008)	0.0009 (0.0002–0.0026)	0.001 (0.0001–0.007)

⁎ Total allele number was 282,912

⁎⁎ Total allele number was 129,206

⁎⁎⁎ Total allele number was 44,324 ACP, Aceruloplasminemia; CI, confidence interval; CoPAN, COASY protein-associated neurodegeneration; FAHN, Fatty acid hydroxylase-associated neurodegeneration; gnomAD, genome Aggregation Database; JES, Jaberi-Elahi syndrome; KRS, Kufor-Rakeb syndrome; LKDMN, Leukoencephalopathy with dystonia and motor neuropathy; MPAN, Mitochondrial membrane protein-associated neurodegeneration; NBIA, Neurodegeneration with brain iron accumulation; NBIA 7, Neurodegeneration with brain iron accumulation 7; NBIA 8, Neurodegeneration with brain iron accumulation 8; PKAN, Panthothenate kinase-associated neurodegeneration; PLAN, Phospholipase A2-associated neurodegeneration; SPG 50, Spastic paraplegia 50, autosomal recessive; WSS, Woodhouse-Sakati syndrome.

**Figure 1:**
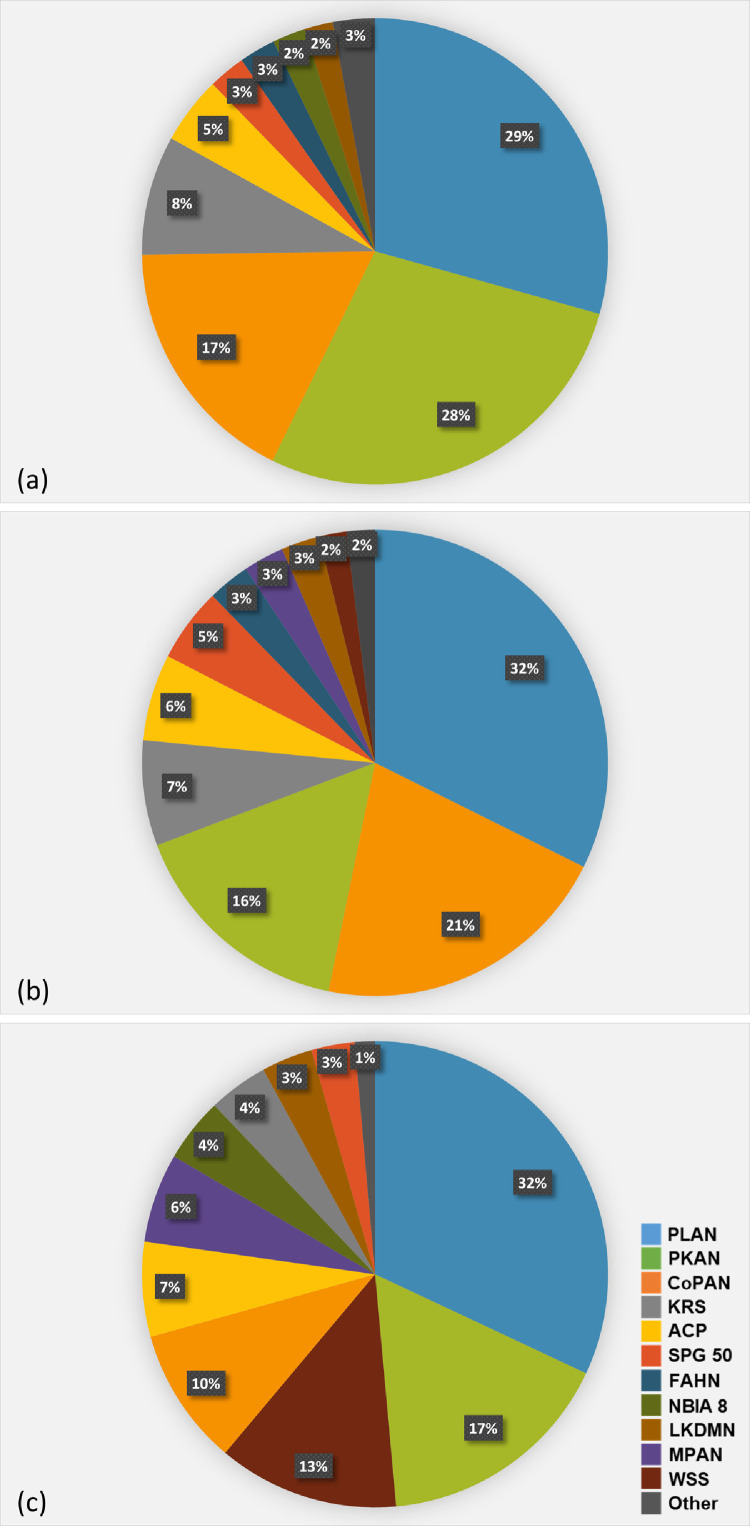
Distribution of NBIA subtypes in the worldwide gnomAD dataset (a), European (non-Finnish) gnomAD dataset (b) and the in-house database (c). The distribution is based on a calculation of lifetime risk per 100,000 based on the allele frequency of 1059 variants in a total of 13 NBIA genes associated with autosomal recessive NBIA disorders. Each colour represent a specific disorder as shown in the legend on the bottom right corner. The section “Other” is comprised of disorders with a lifetime risk below 0.017 per 100,000. ACP, Aceruloplasminemia; CoPAN, COASY protein-associated neurodegeneration; FAHN, Fatty acid-hydroxylase-associated neurodegeneration; gnomAD, genome Aggregation Database; KRS, Kufor-Rakeb syndrome; LKDMN, Leukodystrophy with dystonia and motor neuropathy; MPAN, Mitochondrial membrane protein-associated neurodegeneration; NBIA, Neurodegeneration with brain iron acumulation; NBIA 8, Neurodegeneration with brain iron acumulation 8; PKAN, Panthothenate kinase-associated neurodegeneration; PLAN, Phospholipase A2-associated neurodegeneration; SPG 50, Spastic paraplegia 50, autosomal recessive; WSS, Woodhouse-Sakati syndrome.

**Figure 2:**
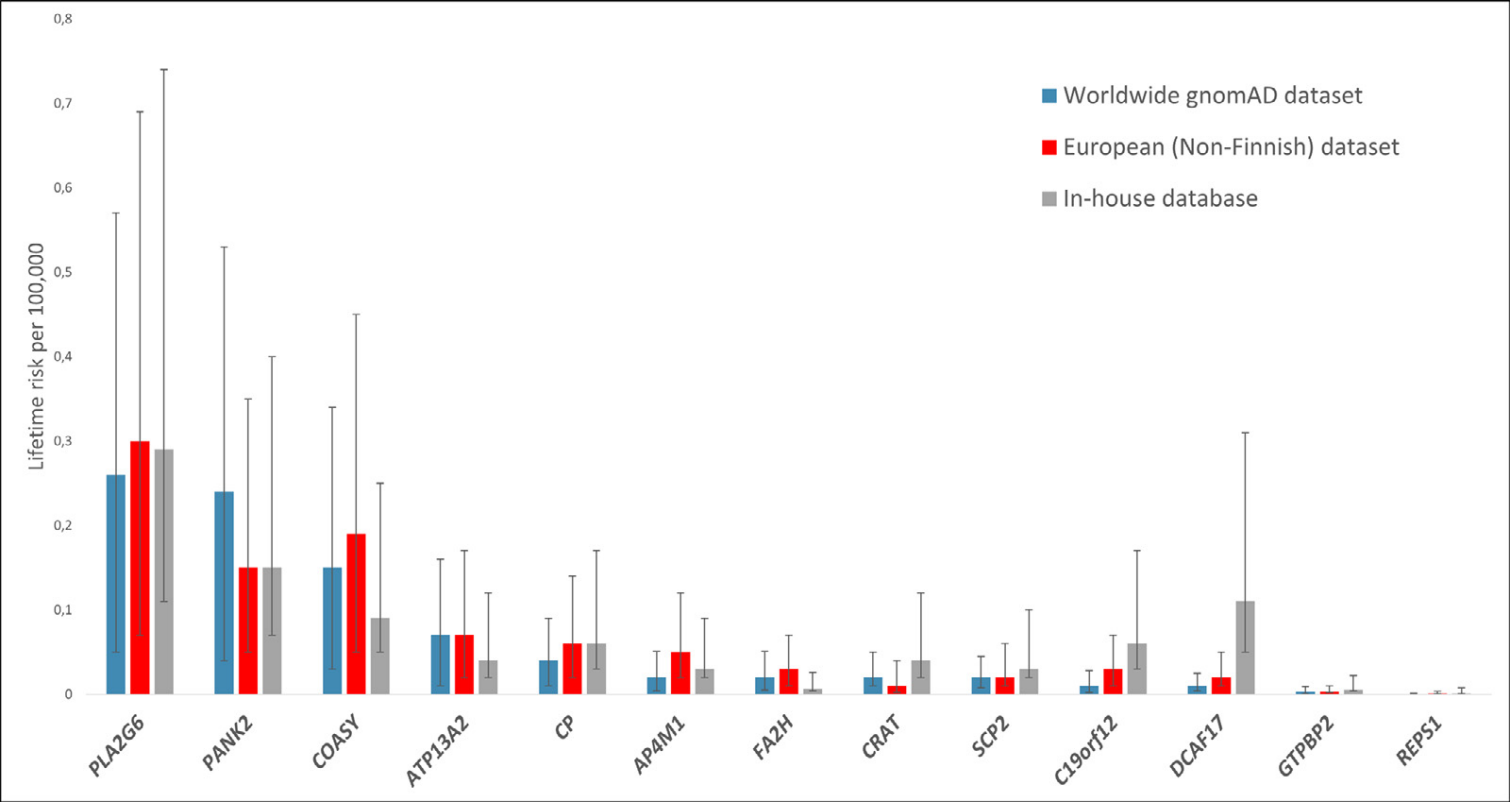
Calculated lifetime risk per 100,000 based on the frequency of 1059 disease-causing alleles in all 13 genes associated with autosomal recessive NBIA disorders. Its comparison among the worldwide gnomAD dataset, European (non-Finnish) dataset and the in-house database is provided. Error bars represent 95%-confidence intervals. gnomAD, genome Aggregation Database; NBIA, Neurodegeneration with brain iron accumulation.

The combined estimated lifetime risk of all 13 investigated NBIA disorders was quite similar when calculated according to global gnomAD (0.88 per 100,000), European (non-Finnish) gnomAD (0.92 per 100,000), and in-house allele frequencies (0.90 per 100,000). The lifetime risks of individual NBIA disorders, however, differed in some aspects in the respective datasets. The highest lifetime risk according to all three datasets was found for disorders associated with biallelic *PLA2G6* variants, ranking before *PANK2* and *COASY* in the global gnomAD dataset, before *COASY* and *PANK2* in the European (non-Finnish) gnomAD dataset, and before *PANK2, DCAF17* and *COASY* in our in-house database, respectively (Figures 1, 2).

Pathogenic variants in *PLA2G6* cause Phospholipase A2-associated neurodegeneration (PLAN) ‒ a disease spectrum ranging from a rapidly progressive and devastating form, infantile neuroaxonal dystrophy (INAD, MIM # 256600), to an atypical neuroaxonal dystrophy (ANAD, MIM # 610217), to young adult manifestations of parkinsonism with or without dystonia and other neurological symptoms.^1^^,^^10^^,^^19^^,^^20^ The combined frequency of *PLA2G6* (likely) pathogenic alleles was 0.0016 in the global gnomAD dataset, 0.0017 in the European (non-Finnish) dataset, and 0.0017 in the in-house database, leading to a calculated lifetime risk of 0.26, 0.30, and 0.29 per 100,000, respectively (Table 2).

The second most common NBIA disorder calculated, as based on the global gnomAD population and the in-house database, was PKAN caused by biallelic variants in *PANK2* (Figures 1, 2, Table 2). *PANK2* encodes the panthotenate kinase enzyme, catalyzing the first step in CoA synthesis.^21^ PKAN (MIM # 606157) has a variable phenotype with two forms: (i) early-onset (classical) form with gait abnormality and progressive dystonia, usually manifesting before the age of 6 years, and (ii) later-onset atypical form with variable presentation and slower progression.^1^ The total frequency of pathogenic *PANK2* alleles was 0.0016 in the global, 0.0012 in the European (non-Finnish) gnomAD dataset and 0.0012 in the in-house database, resulting in lifetime risk estimates of 0.24, 0.15, and 0.15 per 100,000, respectively (Table 2). The calculated lifetime risk of PKAN was higher in East Asian (1.73/100,000) and South Asian populations (0.60 per 100,000) (Suppl. Table 1).

Interestingly, and against all previous assumptions, COASY protein-associated neurodegeneration (CoPAN; MIM # 615643) was among the most frequent disorders. *COASY* encodes for CoA synthase, catalysing the last two steps of CoA synthesis.^1^^,^^22^ The total allele frequency of pathogenic *COASY* alleles was 0.0012 in the global and 0.0014 in the European (non-Finnish) gnomAD dataset, and 0.0009 in the in-house database, resulting in lifetime risk estimates of 0.15, 0.19, and 0.09 per 100,000, respectively (Table 2).

Another more frequent NBIA disorder form according to our in-house database was Woodhouse-Sakati syndrome (WSS, MIM # 241080) due to biallelic *DCAF17* variants. Besides extrapyramidal symptomatology and cognitive impairment, affected individuals with WSS also exhibit hypogonadism, diabetes mellitus, alopecia and cardiac dysrhythmias.^10^ The combined allele frequency of *DCAF17* pathogenic variants was 0.0003 in the global gnomAD, 0.0004 in the European (non-Finnish) gnomAD and 0.0011 in the in-house dataset with consequent calculated lifetime risks of 0.01, 0.02 and 0.11 per 100,000, respectively (Table 2).

Lifetime risks between 0.04 and 0.07 per 100,000 in all datasets were found for Kufor-Rakeb syndrome (KRS; MIM ID # 606693) and aceruloplasminemia (ACP; MIM ID # 604290) based on pathogenic variants in *ATP13A2* and *CP*, respectively (Figures 1, 2, Table 2). KRS is characterized by early onset parkinsonism, pyramidal signs, altered eye movements and dementia.^1^ Its calculated lifetime risk in the Ashkenazi Jew population was 0.68 per 100,000, which is 18-fold higher than in the global gnomAD and 10-fold higher than in the European (non-Finnish) gnomAD population (Table 2). Only a single pathogenic variant in *ATP13A2,* NM_022089.3: c.3057del; p.(Tyr1020Thrfs*3), with an allele frequency of 0.0026, drives the combined allele frequency in this ethnicity (Suppl. Table 1). On the contrary, ACP has the highest frequency in Japan. It is clinically characterized by adult-onset movement disorder, diabetes mellitus, retinal degeneration and dementia.^23^^,^^24^ Its lifetime risk was highest in the East Asian population (0.31 per 100,000) which is almost 8-fold and 5-fold higher, than in the global and European (non-Finnish) gnomAD population, respectively.

The 8 remaining genes had in both the global and the European (non-Finnish) gnomAD population only very low allele frequencies and were altogether responsible for a lifetime risk of 0.11 and 0.16 per 100,000, respectively. It is of interest that MPAN (MIM # 624298) had, according to our results, a rather low lifetime risk in all datasets (<0.06 per 100,000; Table 2). A total of 58 (likely) pathogenic variants in *c19orf12* were verified in the literature, the gnomAD dataset and our in-house database, but a total of 13 variants were excluded due to their reported autosomal dominant inheritance and 1 due to its uncertain significance in ClinVar (Suppl. Table 3). Disorders with the lowest lifetime risk were caused by only recently discovered *GTPBP2* and *REPS1* mutations (both below <0.005 per 100,000 in all datasets; Table 2).

### Calculating the estimated lifetime risk in different populations based on LoF variants

There is a chance of bias as some of the ancestral backgrounds are not well represented in clinical databases. In order to support our results, we estimated lifetime risks by including only LoF variants (namely nonsense, frameshift and splice variants) in the calculations as these are considered disease-causing regardless of a listing in any mutation database. Calculated lifetime risk based on LoF variants was very similar in European (non-Finnish) and global gnomAD population (Suppl. Table 2). Moreover, the distribution of the 5 most common NBIA disorders based on LoF variants corresponded approximately to distribution based on all variants in both global and European (non-Finnish) populations. Relatively high calculated lifetime risks were observed in African and Ashkenazi Jew populations (Suppl. Table 2) although the number of exomes represented in gnomAD was the lowest in these populations. This may be explained by underreporting of missense variants in these populations. We assume that the actual lifetime risks of most NBIA disorders therefore closer resemble the calculations based on the global and European (non-Finnish) gnomAD dataset.

### Disease-causing variants

**Figure 3:**
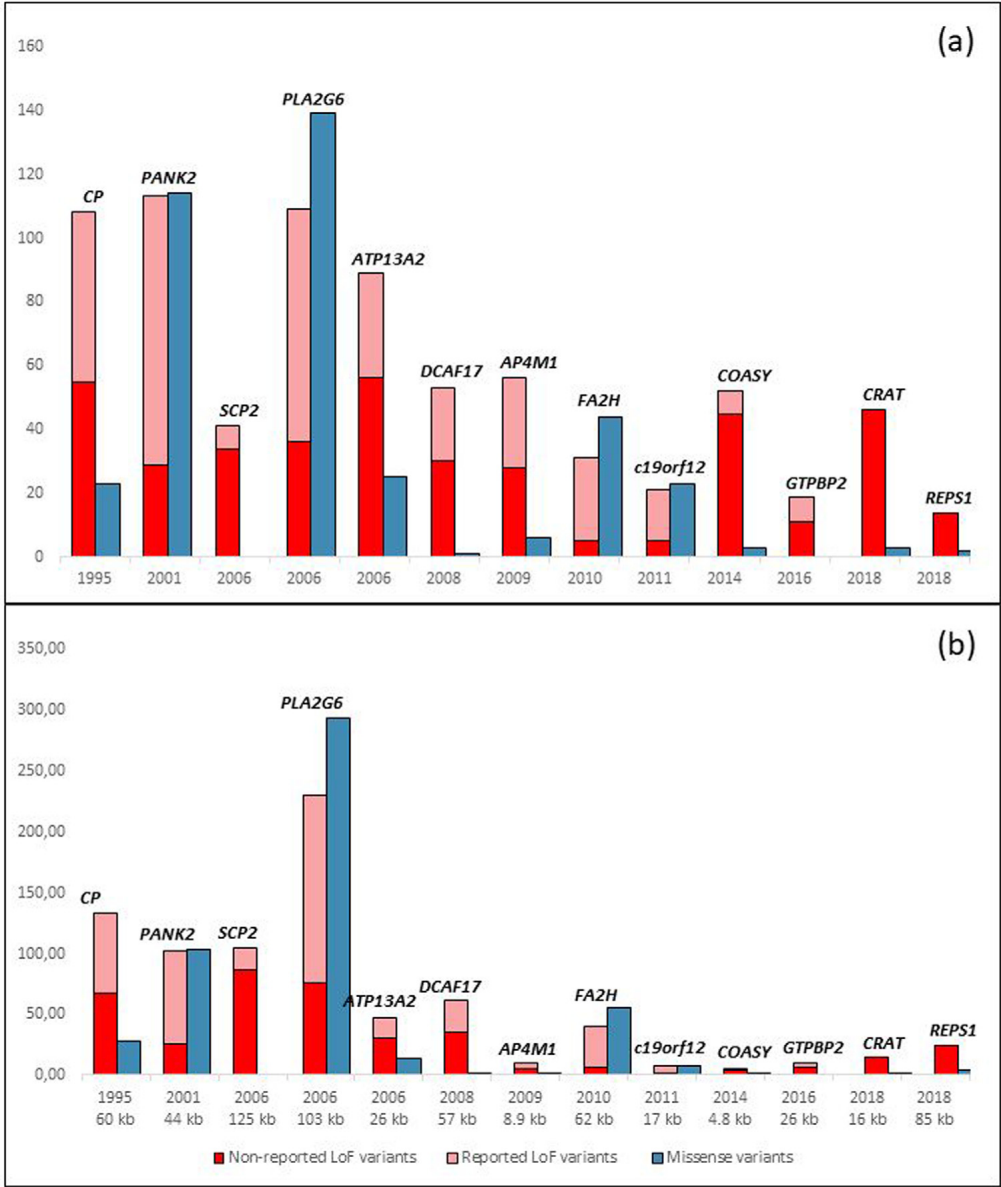
Distribution of both reported and non-reported loss of function, and missense variants in all 13 studied NBIA genes. (a) depicts a correlation between the number of reported variants and a year of gene identification and association with an NBIA disorder. (b) shows a correlation between the number of variants that was adjusted with a factor for the gene size (number of amino acids of a gene x/average number of amino acids of all 13 NBIA genes) and the time period since the identification and association with an NBIA disorder (i.e. 2021 - the year of publication). Due to the multivariate testing, *p*-value of less than 0.01 was considered statistically significant (Spearman correlation coefficient, Bonferroni correction for 5 hypotheses). For each gene, a year of description and/or the size of the gene in kb is shown below (A, B).^19^^,^^22^^,^^34^, ^35^, ^36^, ^37^, ^38^, ^39^, ^40^, ^41^, ^42^, ^43^ Data are expressed as whole numbers. LoF, Loss of Function; NBIA, Neurodegeneration with Brain Iron accumulation; *n*, number.

Distribution of both reported and non-reported LoF and missense variants in all studied NBIA genes is demonstrated in Figure 3. Genes are ordered by the year of identification and association with an NBIA disorder. There was a significant correlation (*R* = 0.7248, *p* = 0.0051; Spearman correlation coefficient) between the number of all disease-causing variants used for the analysis and the time period since the identification and association with an NBIA disorder (Suppl. Table 4, Figure 3a). Moreover, there was a significant correlation between the year of publication and both the number of all reported variants (i.e. reported LoF variants together with all missense variants), and the number of reported LoF variants only (Suppl. Table 4, Figure 3). Similar findings were observed when the number of variants was adjusted with a factor for the gene size (number of amino acids of a gene x/average number of amino acids of all 13 NBIA genes (Suppl. Table 4, Figure 3b).

### Population based approach helps to genetically characterize NBIA disorders

Plotting the number of previously reported LoF and missense alleles in comparison to the number of LoF variants in gnomAD (Figure 3) revealed that no single LoF variant was listed in ClinVar or HGMD as “pathogenic” in *CRAT* and *REPS1* and that *COASY* is depleted for previously reported LoF variants raising the hypothesis that biallelic LoF variants could be incompatible with life and that disease causing alleles have a hypomorphic effect.

## Discussion

This study provides genetically determined lifetime risks for all 13 autosomal recessive NBIA disorders across seven major ethnicities. The combined NBIA lifetime risk derived from both known and predicted pathogenic variants is up to 0.92 per 100,000. We have highlighted that some ethnicities might be at greater risk of developing a specific NBIA disorder, whereas others may have lower genetically predicted lifetime risk. Our study found that the three most common autosomal recessive forms worldwide, but also in European (non-Finnish) ancestral backgrounds are PLAN, PKAN and CoPAN, altogether responsible for up to 75% of the combined overall NBIA lifetime risk (Figures 1, 2, Table 2). Regarding individual NBIA disorders, the one with the highest lifetime risk in all three main datasets was PLAN, being responsible for approx. 30% of the total NBIA lifetime risk, followed by PKAN in the global gnomAD and the in-house database, and by CoPAN in the European (non-Finnish) dataset. Comparable lifetime risks of PKAN in different populations were observed when a similar bioinformatic approach was used by Brezavar and Bonnen^7^ (Suppl. Table 1).

Previous estimates of NBIA distribution are scarce and heterogenous, usually based on period prevalences in local registries.^10^^,^^11^^,^^25^ According to the North American Database analysis from 2015, the four most common NBIA forms were PKAN, BPAN, PLAN and MPAN, with PKAN accounting for half and PLAN for 20% of all NBIA patients.^10^ However, a recent report from Oregon Health & Science University showed a very similar representation of both PKAN and PLAN patients,^25^ which is similar to our findings. As prevalence is a function of both incidence and the duration of the disease, the differences from previous estimates on NBIA distribution may be partly explained by different survival among specific NBIA types (Figure 1). However, accurate comparison with previous estimates could not be made due to the lack of age-stratified epidemiological data.

The lifetime risk of MPAN was surprisingly low in all three main studied datasets (Table 2). The most common variant in our study was the NM_001031726.3: c.204_214del; p.(Gly69Argfs*10) with combined allele frequency of 0.0001 in the global gnomAD population, 0.0002 in the European population and 0.0002 in the in-house database. This variant was also the most common in a cohort of 17 Russian MPAN patients, where it accounted for as much as 73.5% of all pathogenic alleles.^8^ After normalization of the c.204_214del allele frequency in 14,433 exome analyses to the frequency of other pathogenic alleles, the lifetime risk of MPAN in Russia was estimated to be 0.16 per 100,000.^8^ This is more than 13- and 5-fold higher than our calculations based on the worldwide and European gnomAD datasets, respectively, which could be due to a founder effect of the variant c.204_214del.

There was a strong correlation between the number of reported variants and the year of gene identification and association with an NBIA disorder (Figure 3, Suppl. Table 4). Of note, the number of reported variants did not correspond to the calculated lifetime risk, only their combined allele frequency. Although the vast majority of mutations in *REPS1, CRAT* and *COASY* were non-reported LoF variants, the lifetime risk of CoPAN was the third largest in the global population, and in the European (non-Finnish) population, it exceeded PKAN (Figures 1, 3). Given the depletion of reported LoF alleles, we hypothesize that biallelic LoF variants in these genes are incompatible with life. This theory is strengthened by the fact that knockout of *coasy* is incompatible with life in mice.^26^ In addition, a single report described four patients with biallelic LoF *COASY* variants affecting the C-terminus of the protein and prenatal onset of pontocerebellar hypoplasia, microcephaly and arthrogryposis.^27^ In one case, where pregnancy was not early terminated, a boy was born, but died by the age of first month.^27^ Similarly, we believe that complete loss of REPS1 and CRAT are equally prenatally lethal even though data from mouse models or prenatally diagnosed cases are missing. Thus, variants in *COASY, REPS1* and *CRAT* that are causing NBIA are thought to be of hypomorphic nature and might be druggable. These data highlight the potential of population-based approaches in understanding the genetic bases of monogenic disorders. At the same time, they indicate the limitations of our approach as the estimation of the lifetime risk calculated from genetic databases actually represents the lifetime risk from a single-cell zygote stage. Second, population-based observational studies might not reflect a true disease occurrence as some of the NBIA disorders may be common but lead to significant prenatal morbidity and incompatibility with postnatal survival.

It is important to note that in the case of ultra-rare diseases, a single variant may drive the resulting frequency. This is the case for *PANK2* NM_153638.3: c.1133A>G; p.(Asp378Gly) in East Asian population that increases the lifetime risk up to 1.73 per 100,000 which is higher than in the European (non-Finnish) population (Suppl. Table 1). The pathogenicity of this variant is well-documented in three Chinese patients with focal dystonia, radiological signs of NBIA and compound heterozygous *PANK2* mutations.^28^^,^^29^ Allele frequency of c.1133A>G in East Asian gnomAD population, ExAC-East Asian and 1000 Genomes is 0.0032, 0.0037 and 0.0004, respectively, suggesting a founder effect in East Asian ancestry. Nevertheless, given its high frequency, the c.1133A>G would be a plausible candidate for premarital screening in the Chinese/East Asian population. In contrast to our findings, a 12-fold lower lifetime risk (0.16 per 100,000) when compared to our study was found using a similar approach although this particular variant was included in the analysis.^7^ There seems to be some discrepancy since the isolated lifetime risk of a homozygous c.1133A>G in *PANK2* is 0.99 per 100,000. Unfortunately, it could not be clarified, since raw data from Ref.^7^ are not at disposal.

Several important aspects and limitations must be considered when interpreting our results.

(i) As mentioned above, our methodological approach can be applied only in autosomal recessive disorders. Hence the lifetime risks of X-linked BPAN and autosomal dominant neuroferritinopathy could not be assessed.. The same is true for recent reports of dominant inheritance of previously exclusively autosomal recessive disorders.^30^^,^^31^ Our method of estimating the lifetime risk of a genetic disorder cannot be applied to autosomal dominant disorders as population databases would be depleted of their pathogenic variants.
(ii) Underestimation of the lifetime risk may have occurred due to exclusion of variants non-identifiable by whole exome sequencing, such as large genomic rearrangements, variants in flanking intronic regions that are involved in splicing, or epigenetic modifications. Similarly, not all pathogenic variants could have been identified by our approach, since only previously published variants, variants predicted pathogenic by ClinVar and defined LoF variants were considered. This is different from the work of Brezavar and Bonnen, where the pathogenicity was also assessed by four types of online predictors of pathogenicity.^7^ However, as we preferred to stay close to a minimal calculated lifetime risk, we used a more conservative model.
(iii) Moreover, we have shown that the number of reported variants is a function of time period since the first association of a gene with a specific NBIA disorder. It implies that calculated lifetime risks of recently described genes may be falsely low due to minor reporting to population genetic databases.
(iv) On the other hand, overestimation may have occurred when benign variants are falsely interpreted as pathogenic. We assumed that the subgroup of variants of uncertain significance that are in fact disease-causing would compensate for the proportion of likely pathogenic variants that are benign polymorphisms.^12^
(v) The lifetime risk calculated in this study does not account for the incomplete penetrance of variants, as penetrance data concerning NBIA disorders are lacking. However, reduced penetrance is mostly evident in autosomal dominant disorders, although it can occasionally occur in autosomal recessive disorders.^32^
(vi) In addition, our results could be influenced by the phase of variants. As the cis/trans position is only rarely reported in publicly available databases, this factor could not be considered by the present study. However, complex alleles seem to be rare in other recessive disorders^33^ and, therefore, would not most likely affect our results.
(vii) Most importantly, and as mentioned above, estimation of the lifetime risk calculated from genetic databases actually represents the lifetime risk at the moment of conception. Accordingly, some of the NBIA disorders may be common but lead to significant prenatal morbidity and incompatibility with postnatal survival. This may partly explain the differences between calculated cumulative incidence in our study and previous population-based epidemiological reports, such as in the case of CoPAN.

In summary, we provide an estimation of the lifetime risk of all known autosomal recessive NBIA disorders based on population genotypes. The combined lifetime risk estimate was up to 0.92 per 100,000 which by far exceeds previous population-based epidemiological investigations. Moreover, we could rank all 13 disorders by their incidence and define the disorders with the highest lifetime risk as being caused by mutations in *PANK2, PLA2G6* and *COASY*.

## Contributors

HK, data collection, analysis and interpretation, literature search, figures and tables design and preparation, manuscript preparation; JT, statistical treatment of the data; TS, critical review of the manuscript, TM, critical review of the manuscript; TK and MW, conception and study design, critical review of the manuscript, article submission. HK, MW and TK have verified the underlying data. All authors have read and approved the final version of the manuscript.

### Data sharing

Individual participant data will not be made available. Aggregate data, specifically the final list of variants that were rated as “pathogenic” and “likely pathogenic” and the respective number of carriers within the gnomAD dataset and the Munich in-house database will be made available. No other documents will be made available. Anyone who wishes to access the data for any purpose can access the data immediately following publication without an end date from Figshare (https://doi.org/10.6084/m9.figshare.16640281.v2).

## Declaration of interests

HK received a research scholarship from Alzheimer Foundation Czech Republic.

TK served as coordinating investigator of the FORT trial and received research funding from Retrophin, Inc.; served as coordinating investigator of the deferiprone in PKAN randomized and extension trial and received research funding from ApoPharma Inc.; received support from the European Commission 7th Framework Programme (FP7/2007-2013, HEALTHF2-2011; Grant Agreement No. 277984, TIRCON) and from the European Reference Network for Rare Neurological Diseases (ERN-RND), co-funded by the European Commission (ERN-RND: 3HP 767231); provided consulting services to CoA Therapeutics, Comet Therapeutics, and Retrophin, Inc.; received travel support from ApoPharma Inc.

All other authors do not report any conflicts of interest.

## Appendix Supplementary materials

Image, application 1

Image, application 1

Image, application 1

Image, application 1

Image, application 1
