# Supplementary material for: Lifetime risk of autosomal recessive neurodegeneration with brain iron accumulation (NBIA) disorders calculated from genetic databases
Source: eBioMedicine. 2022 Feb 15;77:103869. doi: 10.1016/j.ebiom.2022.103869 (PMC8856992; doi:10.1016/j.ebiom.2022.103869)
Supplement: Supplementary file 4 [file mmc4.docx]

|  | **All variants** | **LoF variants reported** | **LoF non-reported** | **Missense variants** | **All reported variants** |
| --- | --- | --- | --- | --- | --- |
| **Correlation coefficient** | 0.7248 | 0.8183 | 0.3878 | 0.4708 | 0.7386 |
| **p-value** | 0.0051(*) | 0.0006(*) | 0.1904 | 0.1044 | 0.0039(*) |
|  | **All variants^**^** | **LoF variants reported^**^** | **LoF non-reported^**^** | **Missense variants^**^** | **All reported variants^**^** |
| **Correlation coefficient** | 0.7497 | 0.8615 | 0.6501 | 0.5208 | 0.8518 |
| **p-value** | 0.0032(*) | 0.0002(*) | 0.0162 | 0.0680 | 0.0002(*) |
