# Supplementary material for: Lifetime risk of autosomal recessive neurodegeneration with brain iron accumulation (NBIA) disorders calculated from genetic databases
Source: eBioMedicine. 2022 Feb 15;77:103869. doi: 10.1016/j.ebiom.2022.103869 (PMC8856992; doi:10.1016/j.ebiom.2022.103869)
Supplement: Supplementary file 5 [file mmc5.docx]

**Caption for supplementary material**

**Suppl. Tab. 1.** Lifetime risk of 13 autosomal recessive NBIA disorders according to eight subpopulations from the gnomAD dataset and the in-house database (including 95% confidence intervals).

Legend:

ACP, Aceruloplasminemia; CI, confidence interval; CoPAN, COASY protein-associated neurodegeneration; FAHN, Fatty acid hydroxylase-associated neurodegeneration; gnomAD, genome Aggregation Database; JES, Jaberi-Elahi syndrome; KRS, Kufor-Rakeb syndrome; LKDMN, Leukoencephalopathy with dystonia and motor neuropathy; MPAN, Mitochondrial membrane protein-associated neurodegeneration; NBIA, Neurodegeneration with brain iron accumulation; NBIA 7, Neurodegeneration with brain iron accumulation 7; NBIA 8, Neurodegeneration with brain iron accumulation 8; PKAN, Panthothenate kinase-associated neurodegeneration; PLAN, Phospholipase A2-associated neurodegeneration; SPG 50, Spastic paraplegia 50, autosomal recessive; WSS, Woodhouse-Sakati syndrome

**Suppl. Tab. 2.** Lifetime risk of 13 autosomal recessive NBIA disorders calculated based on loss of function variants according to eight subpopulations from the gnomAD dataset and the in-house database (including 95% confidence intervals).

Legend:

ACP, Aceruloplasminemia; CI, confidence interval; CoPAN, COASY protein-associated neurodegeneration; FAHN, Fatty acid hydroxylase-associated neurodegeneration; gnomAD, genome Aggregation Database; JES, Jaberi-Elahi syndrome; KRS, Kufor-Rakeb syndrome; LKDMN, Leukoencephalopathy with dystonia and motor neuropathy; LoF, Loss of Function; MPAN, Mitochondrial membrane protein-associated neurodegeneration; NBIA, Neurodegeneration with brain iron accumulation; NBIA 7, Neurodegeneration with brain iron accumulation 7; NBIA 8, Neurodegeneration with brain iron accumulation 8; PKAN, Panthothenate kinase-associated neurodegeneration; PLAN, Phospholipase A2-associated neurodegeneration; SPG 50, Spastic paraplegia 50, autosomal recessive; WSS, Woodhouse-Sakati syndrome

**Suppl. Tab. 3.** List of variants excluded from the analyses due to their questionable pathogenicity or reported autosomal dominant inheritance.

Legend:

AD, autosomal dominant; gnomAD, genome Aggregation Database

**Suppl. Tab. 4.** Correlation between number of disease-causing variants and the time period since identification and association with an NBIA disorder (i.e. 2021 - year of publication; Spearman correlation coefficient, Bonferroni correction for 5 hypotheses). The number of variants is listed as both non-adjusted and adjusted by a factor of gene size (number of amino acids for gene x/average number of amino acids of 13 NBIA genes).

Legend:

(*) significance on 0.01 level ().

^**^Adjusted by a factor of gene size

LoF, Loss of Function
